# The impact of exercise intensity on whole body and adipose tissue metabolism during energy restriction in sedentary overweight men and postmenopausal women

**DOI:** 10.14814/phy2.13026

**Published:** 2016-12-30

**Authors:** Jean‐Philippe Walhin, Natalie C. Dixon, James A. Betts, Dylan Thompson

**Affiliations:** ^1^ Department for HealthUniversity of BathBathUK

**Keywords:** Adipose tissue, energy restriction, exercise intensity, gene expression, metabolism

## Abstract

This study aimed to establish whether vigorous‐intensity exercise offers additional adipose‐related health benefits and metabolic improvements compared to energy‐matched moderate‐intensity exercise. Thirty‐eight sedentary overweight men (*n* = 24) and postmenopausal women (*n* = 14) aged 52 ± 5 years (mean ± standard deviations [SD]) were prescribed a 3‐week energy deficit (29302 kJ∙week^−1^) achieved by increased isocaloric moderate or vigorous‐intensity exercise (+8372 kJ∙week^−1^) and simultaneous restricted energy intake (−20930 kJ∙week^−1^). Participants were randomly assigned to either an energy‐matched vigorous (VIG;* n* = 18) or moderate (MOD;* n* = 20) intensity exercise group (five times per week at 70% or 50% maximal oxygen uptake, respectively). At baseline and follow‐up, fasted blood samples and abdominal subcutaneous adipose tissue biopsies were obtained and oral glucose tolerance tests conducted. Body mass was reduced similarly in both groups (∆ 2.4 ± 1.1 kg and ∆ 2.4 ± 1.4 kg, respectively, *P *<* *0.05). Insulinemic responses to a standard glucose load decreased similarly at follow‐up relative to baseline in VIG (∆ 8.6 ± 15.4 nmol.120 min.l^−1^) and MOD (∆ 5.4 ± 8.5 nmol.120 min.l^−1^; *P *<* *0.05). Expression of SREBP‐1c and FAS in adipose tissue was significantly down‐regulated, whereas expression of PDK4 and hormone‐sensitive lipase (HSL) was significantly up‐regulated in both groups (*P *<* *0.05). Thus, when energy expenditure and energy deficit are matched, vigorous or moderate‐intensity exercise combined with energy restriction provide broadly similar (positive) changes in metabolic control and adipose tissue gene expression.

## Introduction

We recently showed that vigorous‐intensity exercise has enormously beneficial effects on systemic and adipose tissue measures of metabolic health when young men were challenged with an energy surplus from combined overfeeding and reduced physical activity (Walhin et al. 2013). Notably, the effects from vigorous‐intensity exercise were evident within subcutaneous adipose tissue even when this depot was actively expanding, and despite additional energy intake to account for the energy expended through exercise (i.e., a standardized surplus). In the opposite scenario of weight loss or maintenance, some studies suggest that vigorous high‐intensity exercise is superior to energy‐matched moderate‐intensity exercise for improving glucose control and insulin action (Kang et al. 1996; Swain and Franklin 2006; Trapp et al. 2008). Thus, we hypothesized that, during energy restriction, vigorous‐intensity exercise would provoke more beneficial systemic and adipose‐related changes than moderate‐intensity exercise.

Many intervention studies have investigated the effects of either dietary restriction (e.g., Fontana et al. 2007; Hammer et al. 2008; Larson‐Meyer et al. 2006; Wolever et al. 2008) or exercise alone (e.g., Balducci et al. 2010; Imayama et al. 2012; Jorge et al. 2011; Thompson et al. 2010) on metabolic and inflammatory outcomes. Improvements in systemic markers of low‐grade inflammation often seem to increase with greater weight/fat loss (Selvin et al. 2007). There is strong scientific support for combining regular physical exercise with an energy restricted diet as the most effective treatment for obesity (Hsueh and Buchanan 1994; Brochu et al. 2000; Poirier and Despres 2001) and in the management of type 2 diabetes (Nathan et al. 2009). Surprisingly, few studies have examined the impact of exercise intensity combined with energy restriction on changes within the primary tissue that responds to an energy deficit (i.e., adipose tissue). However, in support of our hypothesis that vigorous‐intensity exercise would confer additional benefits, there is one report that vigorous‐intensity exercise combined with energy restriction increases hormone‐sensitive lipase (HSL) gene expression, whereas moderate‐intensity exercise combined with energy restriction does not have this effect (You et al. 2012).

The purpose of this study was therefore to investigate whether vigorous‐intensity exercise combined with energy restriction would further improve measures of metabolic health and the expression of key genes within adipose tissue compared to energy‐matched moderate‐intensity exercise.

## Methods

### Participants

Thirty‐eight sedentary men (*n* = 24) and postmenopausal women (*n* = 14) with overweight (BMI >25 kg^.^m^−2^) completed this study. Participants were 45–64‐years old with a mean age of 52 ± 5 years (mean ± SD), and a maximal oxygen uptake (V̇O_2max_) of 31.5 ± 4.5 mL^.^kg^−1.^min^−1^, body mass index (BMI) of 30.5 ± 2.5 kg^.^m^−2^ and physical activity level (PAL; total energy expenditure/resting energy expenditure) of 1.58 ± 0.17. Volunteers were recruited from the local community. Participants had been weight stable (±3 kg) for at least 6 months. Women were required to be postmenopausal in order to take part in the study (no menstruation for at least a year, Witteman et al. 1989). To be eligible, participants were required to report no participation in regular structured exercise and that they did not do >30 min of moderate‐intensity exercise, accumulated in 10 min bouts, on most days of the week. Participants completed a health questionnaire and Physical Activity Readiness Questionnaire (PAR‐Q) to further verify eligibility and provided written and verbal consent prior to taking part. The protocol was approved by the Bath NHS Research Ethics Committee (REC reference number: 08/H0101/194) in accordance with the *Declaration of Helsinki*. Individuals who smoked, suffered from a condition known to interact with the study measures or took regular medication that may have interfered with the results were excluded from the study.

### Experimental design and protocol

A randomized parallel group design was used for this trial (registration number: ISRCTN86152135). Participants were randomly allocated by a third party to experience a fixed energy deficit of 29302 kJ∙week^−1^ (7000 kcal∙week^−1^), induced by energy restriction and increased physical activity via either moderate (MOD, *n* = 20) or vigorous (VIG, *n* = 18) intensity physical exercise for 3 weeks. Volunteers in both groups were asked to reduce their energy intake by under‐consuming their habitual diet in order to create an energy deficit of 20930 kJ^.^week^−1^ (5000 kcal∙week^−1^). In addition, participants in the MOD group increased their physical activity by walking on a treadmill five times per week at 50% of their maximum oxygen uptake while participants in the VIG group increased their physical activity by walking on a treadmill five times per week at 70% of their maximum oxygen uptake. Importantly, both groups expended 8372 kJ^.^week^−1^ (1674 kJ/400 kcal per session for both groups) above rest through increased physical activity. Participants also abstained from tea/coffee and alcohol the day immediately before each trial.

On Day 1, participants arrived at the laboratory at 0700 ± 0.5 h following an overnight fast (≥10 h). Body mass was measured to the nearest 0.1 kg using electronic scales (Tanita Corporation, Japan). Anthropometric measurements were made in triplicate using a metallic tape measure (Lufkin, US) before lean and fat mass were determined using Dual‐Energy X‐ray absorptiometry (DEXA; Discovery, Hologic, Bedford, UK). A baseline blood sample was collected and a subcutaneous abdominal adipose tissue sample was then obtained before an Oral Glucose Tolerance Test (OGTT) was performed. Blood pressure was measured in triplicate at the end of each trial. This entire protocol was then repeated at follow‐up (Day 21).

### Preliminary measurements

Participants recorded their food and fluid intake for a typical 7‐day period prior to taking part in the study using a set of digital weighing scales (Model 3001, Salter, Kent, UK). Dietary records were analyzed using the software CompEat Pro Version 5.8.0 (Nutrition Systems, UK), which is based on food composition tables for UK foods. Energy intake was estimated using this software and Diet Induced Thermogenesis (DIT) was estimated preintervention as 10% of energy intake (Westerterp 2004). Combined heart‐rate/accelerometry (Actiheart^™^) was used to determine habitual physical activity thermogenesis at baseline.

Participants underwent a maximal oxygen uptake test on a treadmill prior to the intervention (Woodway, ELG 70, Weiss, Germany) following a method adapted from Taylor et al. (*1955*). The percentage of O_2_ and CO_2_ in expired air samples was determined using paramagnetic and infrared gas analyzers, respectively (Series 1400, Servomex Ltd., Sussex, UK).

During the days leading up to the first trial, volunteers followed their normal lifestyle and consumed their habitual diet.

### Intervention description and assumptions

The reported 7‐day food and fluid intake from each participant was used to calculate the energy intake required to cause an energy deficit of 20930 kJ^.^week^−1^ (5000 kcal^.^week^−1^) from energy restriction alone. This was achieved by subtracting 20930 kJ^.^week^−1^ (5000 kcal^.^week^−1^) from their recorded total weekly energy expenditure (assessed by the Actiheart^™^ during the diet recall week, Thompson et al. 2006). The dietary energy restriction was based on participants’ energy expenditure as individuals with overweight and obesity have been shown to under‐report food intake (Macdiarmid and Blundell 1998). Based on this, participants' recorded diets were scaled down in order to induce the desired energy deficit during the intervention. This was achieved by multiplying the weight of all individual foods from the 7‐day record by a factor derived from the energy expenditure data in order to achieve the desired energy intake during the intervention. Participants repeated this 1 week diet three times during the intervention.

Volunteers in both groups were required to perform five exercise sessions a week at the correct speed and gradient required for them to achieve either 50% V̇O_2_ max for the MOD group or 70% V̇O_2_ max for the VIG group. A corresponding heart rate (HR) at this gradient and speed was estimated from the relationship between HR and % V̇O_2_ max. Energy expenditure at the desired intensity was utilized to calculate a set distance (km) required to expend 1674 kJ (400 kcal) above resting energy expenditure. In order to account for post‐exercise oxygen consumption, measured oxygen consumption was adjusted by an extra 5.4% for the MOD group and by 6.4% for the VIG group to derive energy expenditure for each exercise bout (Gore and Withers 1990). Participants were asked to complete this distance at predicted HR below 50% V̇O_2max_ for the MOD group or at least 70% V̇O_2max_ for the VIG group. Assuming a linear relationship between speed and energy expenditure when walking at a set gradient (Franklin 2000), participants were then able to adjust the speed in order to achieve the target heart rate and intensity. All participants confirmed they adhered to the exercise training by filling out an exercise log and attending a weekly supervised exercise session. They also confirmed to have only consumed the prescribed foods over the course of the intervention. All participants performed the final exercise bout prescribed 48 h before follow‐up measurements were collected.

### Blood sampling & analysis

Following the application of a topical local anesthetic (1.5 mL Ametop gel, Smith & Nephew, Hull, England) an 18‐gauge 1.3 × 45 mm cannula (BD Venflon Pro) was inserted into an antecubital vein and a baseline blood sample collected. For the OGTT, participants ingested 113 mL (75 g) of glucose (maltodextrin) solution (Polycal, Nutricia, UK) within 5 min. Once ingested, blood samples were collected every 15 min for 2 h. Blood samples were processed and analyzed as previously described (Walhin et al. 2013).

### Adipose tissue biopsy and processing

Adipose tissue biopsies were performed under local anesthesia (1% lidocaine) after insertion of the cannula and before the OGTT. Subcutaneous abdominal adipose tissue was biopsied 4–7 cm lateral of the umbilicus with a 14‐gauge needle using the needle aspiration technique, with follow‐up biopsies sampled from the opposite side. The sample was cleaned with isotonic saline and any clot was manually removed. After weighing the sample, it was homogenized in 5 mL of Trizol (Invitrogen, UK) and placed on dry ice before being stored at −80°C. Subsequently, samples were defrosted and spun at 2500*g* for 5 min at 4°C. The top layer and pellet were removed and 200 *μ*L of chloroform was added per 1 mL of Trizol. After shaking the mixture vigorously for 15 sec samples were incubated at room temperature for 3 min and then centrifuged at 2500*g* for 5 min at 4°C. The aqueous phase was removed and used for gene expression. Participants were given the option to opt out of this procedure, adipose tissue biopsies were collected from a subset of participants, as a result there are *n* = 12 in both groups.

### Quantitative real‐time PCR

The aqueous phase was mixed with an equal volume of 70% Ethanol before being loaded on an RNeasy mini column for extraction (Qiagen, Crawley, UK) as previously described (Walhin et al. 2013). Assays from Applied Biosystems were used: ADIPONECTIN (Hs00605917_m1), LEPTIN (Hs00174877_m1), SREBP‐1c (Hs01088691_m1), PDK4(Hs00176875_m1), FAS (Hs00188012_m1), PPAR*γ* (Hs01115513_m1), GLUT4 (Hs00168966_m1), IRS2 (Hs00275843_s1), LPL (Hs01012567_m1), HSL (Hs00193510_m1), VISFATIN (Hs00237184_m1), IRS1 (Hs00178563_m1), IL18 (Hs00155517_m1), IL6 (Hs00985639_m1), AMPK (Hs01562315_m1 & Hs00178903_m1 combined). Real‐time PCR was performed using a StepOne^™^ (Applied Biosystems, Warrington, UK). PPIA (Peptidylpropyl isomerase A) was used as an endogenous control. The comparative Ct method was used to process the data where ΔCt = Ct Target gene – Ct Endogenous control. Data were normalized to an internal calibrator and baseline.

### Statistical analysis

To simplify data analysis and facilitate a more meaningful interpretation of an otherwise complex factorial research design (Matthews et al. 1990; Hopkins et al. 2009), serial measurements of glucose and insulin at baseline and follow‐up were converted into simple summary statistics to illustrate the net response of each parameter (i.e., within‐subject peak concentrations, time to peak and incremental area under curve, Wolever and Jenkins 1986). Preplanned contrasts (#) were conducted in relation to the absolute group differences both at final follow‐up and the relative change from baseline (Atkinson 2002). The precise time course of responses within and between trials were analyzed using factorial 2‐ and three‐way mixed‐model analysis of variance (group × day & group × day × time, respectively) irrespective of minor deviations from a normal distribution (Maxwell and Delaney 1990) but with the Greenhouse‐Geisser correction applied to intraindividual contrasts for *ɛ*<0.75 and the Huynh‐Feldt correction adopted for less severe asphericity (Atkinson 2002). Where significant interactions were observed, multiple *t*‐tests were applied to determine the location of variance both between treatments at level time points and between time points within each treatment relative to baseline, with both methods subject to a Holm–Bonferroni correction (Atkinson 2002). For all the above statistical approaches, statistical significance was set at an alpha level of *P *≤* *0.05. Data are presented in text as means and standard deviations (SDs), whereas the variance bars on figures are confidence intervals (CIs) that have been corrected to remove interindividual variation (Masson and Loftus 2003). For reference, the magnitude of these CIs illustrate the change at each time point relative to baseline such that, in general, plotted means whose CIs do not overlap by more than one‐half of one side of an interval are likely to be deemed statistically different according to conventional significance testing (Masson and Loftus 2003).

A main effect of day denotes an effect of energy deficit *per se* (†: Day 1 vs. Day 21 both groups), whereas a day × group interaction means there is a mediating effect of exercise intensity (*). Statistical analysis for the gene expression data was carried out on the log‐transformed data following normalization to a housekeeper, an internal calibrator and baseline (Livak and Schmittgen 2001). Any change score (normalized follow‐up data vs. normalized baseline data) that was over 3 SD from the mean were excluded from the presentation shown in Figure 2 but were included in the statistical analysis. Samples outside the detectable limit (Ct>35) were excluded from the analysis. The Pearson rank correlation was employed to determine the strength of relationships between parameters. For CRP and IL‐6, the mean and standard deviation of the change (∆) were calculated using all data points. Any change score that was over 3 SD away from the mean were excluded from analysis as this would likely have been due to an acute inflammatory response. This is mentioned in the Figure legend when this rule has been applied.

## Results

### Anthropometric and physiological measures

Anthropometric and physiological measures pre‐ and postintervention are summarized in Table 1. Body mass, waist and hip circumference, fat mass, abdominal fat, systolic and diastolic blood pressure significantly decreased (*P *≤* *0.05) in both groups. There was no day × group interaction.

**Table 1:** Anthropometric and physiological characteristics measured before and after 3 weeks of energy restriction with moderate‐intensity or vigorous‐intensity physical exercise

	**MOD**			**VIG**		
	Baseline	Follow‐up	Δ (95% CI)	Baseline	Follow‐up	Δ (95% CI)
Body mass (kg)	90.0 ± 13.6	87.6 ± 13.3	−2.4 (1.7–3.1)	89.5 ± 16.6	87.1 ± 16.6	−2.4 (1.9–2.9)^a^
Waist circumference (cm)	104.7 ± 6.8	102.9 ± 6.5	−1.8 (0.9–2.7)	101.0 ± 10.4	98.8 ± 10.5	−2.2 (0.9–3.4)^a^
Hip circumference (cm)	111.0 ± 5.9	109.3 ± 5.0	−1.6 (0.7–2.8)	110.6 ± 6.2	108.2 ± 5.0	−2.4 (1.4–3.5)^a^
Fat mass (kg; DEXA)	32.3 ± 5.6	30.5 ± 5.0	−1.8 (0.9–2.8)	30.9 ± 5.4	29.0 ± 5.1	−1.9 (1.2–2.6)^a^
Lean mass (kg; DEXA)	54.2 ± 11.4	53.5 ± 11.0	−0.7 (0.4–1.8)	54.8 ± 14.7	54.5 ± 14.9	−0.3 (−1.1–0.5)
Abdominal fat (kg; DEXA)	4.8 ± 1.4	4.4 ± 1.3	−0.4 (4.2–5.5)	4.4 ± 1.1	3.9 ± 1.0	−0.4 (3.8–4.9)^a^
Systolic BP (mmHg)	128 ± 13	121 ± 15	−7 (3–10)	135 ± 11	128 ± 13	−7 (2–12)^a^
Diastolic BP (mmHg)	80 ± 12	75 ± 10	−5 (1–8)	85 ± 9	79 ± 9	−6 (3–9)^a^

Mean ± SD. Change scores shown with 95% confidence intervals.

a Main effect of day (i.e., Day 1 vs. Day 21 both groups; *P *<* *0.001).

### Insulin & glucose responses to OGTT

**Figure 1:**
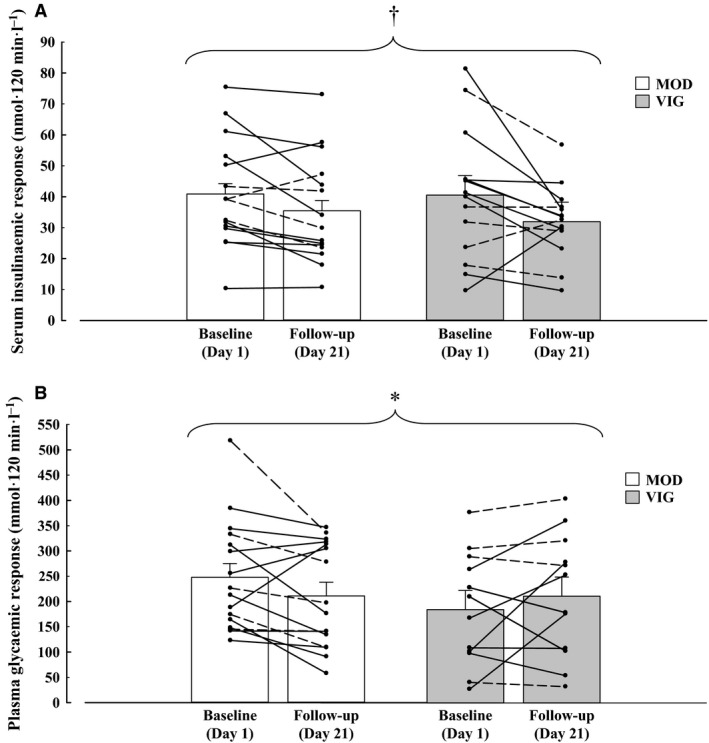
Serum insulin 2 h iAUC (A) for the MOD group (*n* = 15) and the VIG group (*n* = 14), plasma glucose 2 h iAUC (B; means ± CI) for the MOD group (*n* = 16) and the VIG group (*n* = 12) before and after 3 weeks of energy restriction with moderate‐intensity or vigorous‐intensity physical exercise. There were technical problems with the cannula on a few occasions and this explains the reduced sample size for some outcomes. † denotes a main effect of day (*P *=* *0.005) * denotes a dayxgroup interaction (*P *=* *0.04). The dotted line denotes the individual data points from women while the continuous line denotes the individual data points from men.

The insulinemic response following the OGTT was significantly reduced (*P *=* *0.005) at follow‐up for both groups with a decrease in 2 h insulin iAUC as shown by Figure 1. The plasma glucose concentrations in response to the OGTT at follow‐up were unaffected in both groups (see supplemental data Figure S2). The glycemic response following the OGTT decreased in the MOD group while the 2 h venous glucose iAUC modestly increased in the VIG group postintervention (day × group interaction *P *=* *0.04; Figure 1).

The Homeostasis Model Assessment of Insulin Resistance (HOMA‐IR) and the Homeostasis Model Assessment of *β*‐cell function (HOMA‐*β*) decreased (*P *=* *0.001) at follow‐up in both groups. The Matsuda Index or Composite Index (ISI comp) increased (*P *=* *0.007) at follow‐up in both groups. Data is summarized in Table 2.

**Table 2:** HOMA‐IR, HOMA‐β, Composite Index values and fasted blood measurements at baseline and follow‐up

	MOD			VIG		
	Baseline	Follow‐up	Δ (95% CI)	Baseline	Follow‐up	Δ (95% CI)
HOMA‐IR	1.98 ± 1.06	1.65 ± 0.82	−0.33 (−0.17–0.82)	2.36 ± 2.18	1.41 ± 1.45	−0.95 (0.44–1.46)^a^
HOMA‐*β* (%)	99 ± 58	76 ± 33	−22 (−4–48)	84 ± 44	52 ± 19	−31 (13–50)^a^
Insulin sensitivity index (comp)	5.6 ± 3.6	6.1 ± 2.6	0.5 (−1.0–2.1)	5.9 ± 3.3	8.1 ± 3.5	2.2 (1.2–3.3)^a^
Adiponectin (*μ*g mL^−1^)	8.7 ± 4.6	7.9 ± 4.5	−0.8 (0.4–1.3)	8.9 ± 6.2	7.6 ± 4.9	−1.3 (−0.1–2.6)^a^
Leptin (ng mL^−1^)	14.6 ± 9.7	9.2 ± 6.4	−5.4 (3.2–7.6)	14.7 ± 10.6	7.7 ± 6.9	−7.0 (4.0–10.0)^a^
Total cholesterol (mmol L^−1^)	6.1 ± 1.1	5.9 ± 1.0	−0.3 (0.0–0.5)	5.8 ± 1.6	5.2 ± 0.9	−0.6 (0.0–1.1)^a^
HDL cholesterol (mmol L^−1^)	1.5 ± 0.5	1.4 ± 0.4	0.0 (0.0–0.1)	1.5 ± 0.5	1.4 ± 0.4	−0.1 (0.0–0.2)^a^
LDL cholesterol (mmol L^−1^)	4.4 ± 1.0	4.2 ± 0.8	−0.2 (−0.1–0.4)	4.0 ± 1.3	3.6 ± 0.9	−0.4 (−0.1–0.9)^a^
NEFA (mmol L^−1^)	0.6 ± 0.2	0.7 ± 0.1	0.0 (−0.1–0.1)	0.7 ± 0.3	0.8 ± 0.3	0.1 (0.0–0.2)
TAG (mmol L^−1^)	1.5 ± 0.6	1.2 ± 0.4	−0.2 (0.0–0.5)	1.5 ± 1.0	1.3 ± 0.8	−0.3 (0.1–0.5)^a^
ALT (U L^−1^)	32 ± 20	27 ± 13	−5 (−0.6–10.7)	38 ± 21	31 ± 15	−7 (−0.3–14.1)^a^
CRP (mg L^−1^)	2.6 ± 1.7	2.0 ± 1.2	−0.6 (0.0–1.1)	2.2 ± 1.6	1.6 ± 1.4	−0.5 (0.3–0.8)^a^
IL‐6 (pg mL^−1^)	1.6 ± 0.5	1.5 ± 0.5	−0.1 (0.0–0.3)	1.3 ± 0.4	1.4 ± 0.8	−0.1 (−0.2–0.3)
Whole blood WBC (×10^9^ L^−1^)	5.4 ± 1.1	4.8 ± 0.9	−0.6 (0.2–1.0)	5.3 ± 1.2	4.7 ± 1.0	−0.7 (0.2–1.1)^a^

Mean ± SD. Change scores shown with 95% confidence intervals.

a Main effect of day (i.e., Day 1 vs. Day 21 both groups; *P *≤* *0.05).

### Fasted blood measurements

Serum adiponectin, leptin, cholesterol, HDL cholesterol, LDL cholesterol, TAG, ALT, CRP and whole blood WBC concentrations significantly decreased (*P ≤ *0.05) at follow‐up in both groups. Data are summarized in Table 2.

### Adipose tissue gene expression

**Figure 2:**
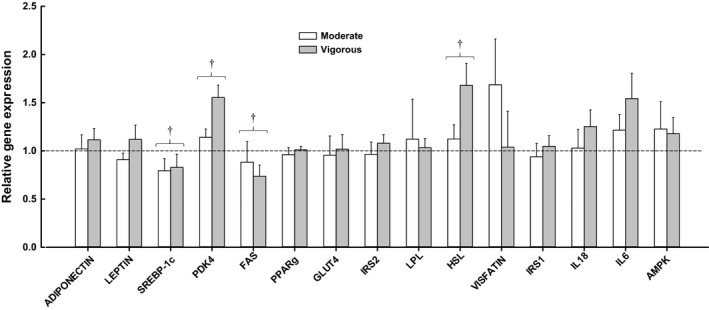
Relative gene expression of several key genes measured in adipose tissue at baseline and follow‐up for the MOD group (*n* = 12) and the VIG group (*n* = 12). Dashed line represents no change. Data normalized to PPIA, baseline, and internal calibrator. Any change score (normalized follow‐up data vs. normalized baseline data) that was over 3 SD from the mean (11 data points out of 360) were excluded from the presentation shown in this Figure, but were included in the statistical analysis which used the logged values. Samples outside the detectable limit (Ct>35; *n* = 8) were excluded from the analysis. FAS (MOD,* n* = 11), IL18 (MOD,* n* = 8), IL6 (MOD,* n* = 9; VIG,* n* = 11). Values are means ± SEM. † main effect of day (i.e., Day 1 vs. Day 21 both groups; *P* ≤ 0.05).

Expression of SREBP‐1c and FAS was significantly down‐regulated (*P *=* *0.004) in both groups. Expression of PDK4 (*P *=* *0.001) and HSL (*P *=* *0.04) was significantly up‐regulated in both groups. There was a trend for a day × group interaction (*P *=* *0.057) in the expression of HSL. Gene expression data are presented in Figure 2.

## Discussion

This study assessed the impact of energy restriction combined with energy‐matched moderate or vigorous‐intensity exercise in sedentary men and postmenopausal women with overweight/obesity. We demonstrated that 3 weeks of energy restriction and increased physical activity improved fasting measures of metabolic control and inflammation, responses to an OGTT, and expression of several key genes within adipose tissue. These positive changes were mostly unaffected by exercise intensity.

In order to investigate whether vigorous‐intensity exercise offers added benefits compared to moderate‐intensity exercise in improving metabolic control and inflammatory markers in sedentary individuals with overweight/obesity, we used a model where energy restriction and physical exercise energy expenditure were carefully matched between groups. This combined approach has been successfully used in several other studies for similar purposes (You et al. 2004; Nicklas et al. 2009). We successfully induced a standardized negative energy balance, resulting in a weight loss of 2.4 ± 1.4 kg and 2.4 ± 1.1 kg in MOD and VIG groups, respectively. Our data suggest that after 3 weeks of energy restriction and increased physical exercise, improvements in insulin sensitivity and metabolic control were evident as illustrated by a similar reduction in waist/hip circumferences, fat mass, blood pressure, total cholesterol and LDL cholesterol, TAG, ALT, CRP, whole blood WBC, and by the improvement in the expression of four key genes expressed within adipose tissue. Notably, the majority of these changes were unaffected by exercise intensity.

Although the benefits of energy restriction combined with increased physical activity on human health are well documented, little is known about the impact on the transcriptome of adipose tissue during energy deficit in humans. The adipose organ is a major site for energy storage and one of the main tissues being impacted during a period of negative energy balance (Capel et al. 2009; Bouchard et al. 2010). SREBP‐1c was down‐regulated to a similar degree in both groups in this study. SREBP‐1c is a transcription factor that regulates the expression of the lipogenic enzyme FAS (Minehira et al. 2003) and our data support this as there was a strong positive correlation between changes in the expression of SREBP‐1c and changes in the expression of FAS (*r* = 0.77; *P *<* *0.001) which was also similarly down‐regulated in both groups. Changes at the mRNA level have been shown to translate to similar changes in the active form of the protein for SREBP‐1c (Repa et al. 2000) and FAS (Claycombe et al. 1998). SREBP‐1c mediates the effects of insulin and glucose on the regulation of key genes associated with glucose metabolism; there was an inverse correlation between changes in insulin iAUC and changes in the expression of SREBP‐1c (*r* = −0.47; *P *=* *0.03). Tsintzas et al. (*2006*) showed that SREBP‐1c mRNA was down‐regulated 2.5 fold in skeletal muscle following 48 h of starvation in healthy males. Conversely, overfeeding has been shown to increase the expression of SREBP‐1c mRNA in the adipose tissue of participants with a range of BMI (Minehira et al. 2004; Walhin et al. 2013). Gene expression of PDK4 in adipose tissue increased in both groups, potentially highlighting a switch of oxidative fuel from glucose to fatty acids by the adipose tissue. Expression of this gene is regulated by glucocorticoids, retinoic acid, and insulin (Kwon and Harris 2004). During starvation, the activation of PDK4 results in a switch of oxidative fuel use from glucose to fatty acids (Wu et al. 2000; Jeoung et al. 2006; Tsintzas et al. 2006). The model used in this study decreased HOMA‐IR, HOMA‐*β* and the insulinemic response to a standard glucose load, while increasing ISI similarly in both groups, potentially favoring a switch to fatty acid metabolism. Hormone‐sensitive lipase (HSL) is an enzyme that regulates adipose tissue lipid metabolism as it catalyzes the hydrolysis of triglycerides and diglycerides (Holm et al. 2000) and plays an important role in chronic exercise‐induced changes in adipose tissue metabolism. Gene expression of HSL in adipose tissue significantly increased in both groups. Changes in HSL mRNA and protein levels have been shown to be correlated (Jocken et al. 2007) and HSL is a major determinant of the maximum lipolytic capacity of human adipocytes (Large et al. 1998). Collectively, our results indicate that combined energy restriction and increased physical activity leads to an increase in the oxidation of fatty acids by adipose tissue independent of exercise intensity (as indicated by changes in the following transcripts: ↓ SRPBP‐1c, ↓ FAS, ↑ PDK4, ↑ HSL).

Short‐term negative energy balance positively impacts metabolic control whether that is induced through energy restriction alone (Weiss et al. 2006; Fontana et al. 2007; Lagerpusch et al. 2011) or exercise alone (Oshida et al. 1989; Kang et al. 1996; Tjonna et al. 2008). A review of epidemiological data by Swain & Franklin (Swain and Franklin 2006) examined the benefits of moderate versus vigorous‐intensity aerobic exercise in improving cardiovascular health and reducing the risk of coronary heart disease. They concluded that if the total energy expenditure of exercise is held constant, exercise performed at a vigorous‐intensity appears to further reduce the risk of cardiovascular disease compared to moderate‐intensity exercise. Conversely, a study by Nicklas et al. (2009) found that the combination of energy restriction and increased physical activity (independent of intensity) produced similar weight loss and changes in lipids, glucose, and insulin metabolism across treatment groups. The present study also suggests that, during energy restriction, the intensity of exercise is less important when exercise energy expenditure is carefully matched.

The results of this study indicate that while vigorous‐intensity exercise has a dramatic positive impact on maintaining metabolic control during a short period of positive energy balance (Walhin et al. 2013), it does not appear to provide profound additional health‐related benefits compared to moderate‐intensity exercise during a negative energy balance. Interestingly, the genes that were up‐regulated (SREBP‐1c & FAS) and down‐regulated (PDK4, HSL) by a period of positive energy balance (Walhin et al. 2013) were down‐regulated (SREBP1c & FAS) and up‐regulated (PDK4, HSL) by a period of negative energy balance in this study. Collectively, these results highlight the adaptability of human adipose tissue during manipulations of energy balance. Although we cannot determine this conclusively based on the current design and available information, we tentatively propose that vigorous‐intensity exercise has a more powerful effect on these genes in the context of an energy surplus and a less powerful effect during an energy deficit. This may be related to the sophisticated and carefully orchestrated mechanisms that have evolved to mount a powerful defense against fat loss (Badman and Flier 2007; Friedman 2009; Oswal and Yeo 2010) and that these systems overwhelm the more subtle impact from differences in exercise intensity during energy restriction.

To conclude, this experiment shows that short‐term energy restriction combined with either vigorous or moderate‐intensity physical exercise improves insulin sensitivity, lipid profiles, and markers of inflammation. The expression of several key genes in adipose tissue of sedentary men and postmenopausal women with overweight/obesity was also positively and similarly altered with either vigorous or moderate‐intensity exercise when energy expenditure and energy deficit were matched. Thus, the benefits of increased physical exercise combined with energy restriction are mostly independent of exercise intensity.

## Conflict of Interest

The authors declare no conflict of interest.
